# Formal education, health literacy and Mini-Mental State Examination

**DOI:** 10.1590/S1980-57642011DN05010005

**Published:** 2011

**Authors:** Sonia Maria Dozzi Brucki, Letícia Lessa Mansur, Maria Teresa Carthery-Goulart, Ricardo Nitrini

**Affiliations:** 1 Behavioral and Cognitive Neurology Unit, Department of Neurology, and Cognitive Disorders Reference Center (CEREDIC). Hospital das Clínicas of the University of São Paulo School of Medicine, São Paulo SP, Brazil.; 2 Department of Physiotherapy, Speech Therapy and Occupational Therapy, University of São Paulo, São Paulo SP, Brazil.

**Keywords:** literacy, health literacy, functional literacy, S-TOFHLA, education, Mini-Mental State Examination

## Abstract

The Mini-Mental State Examination (MMSE) is a widely used screening test for cognitive impairment, but is heavily biased by education. Educational level has frequently been ranked using years of schooling, which may not be a good indirect measure of educational level because there is great heterogeneity in standards of schooling among populations and across regions of the same country. S-TOFHLA is a measure of health literacy with some results indicating that it is a good measure for literacy level.

**Objective:**

To evaluate the correlations between years of schooling and scores on the S-TOFHLA and the MMSE.

**Methods:**

Healthy subjects without cognitive impairment were submitted to the S-TOFHLA and the MMSE. Correlations and regression analysis were performed to determine possible associations among variables.

**Results:**

Both years of schooling and S-TOFHLA scores were strongly correlated with MMSE scores, but the strongest association was reached by the S-TOFHLA (r=0.702, p<0.01), where the S-TOFHLA was the best predictor of MMSE scores (R^2^=0.494, p<0.001).

**Conclusions:**

A stronger association between S-TOFHLA scores and MMSE performance was found than between years of education and MMSE scores. This finding justifies further studies incorporating years of schooling together with S-TOFHLA score, to evaluate cognitive performance.

## Abstract (pt)

O Mini-Exame do Estado Mental (MEEM) é um teste de rastreio para comprometimento cognitivo largamente utilizado, porém, é fortemente influenciado pela educação. Nível educacional tem sido frequentemente avaliado usando-se o nível de escolaridade, o que pode não ser uma boa medida indireta de nível educacional, porque existe uma grande heterogeneidade entre a escolarização entre populações e mesmo entre regiões de um mesmo país. S-TOFHLA é uma medida de alfabetismo em saúde, com alguns resultados indicando que seja uma boa medida para nível de alfabetismo.

**Objetivos:**

Avaliar as correlações entre anos de escolarização e os escores no S-TOFHLA e MEEM. Correlações e análise de regressão foram realizadas para determinar possíveis associações entre as variáveis.

**Resultados:**

Ambos, anos de escolarização e escores no S-TOFHLA, foram fortemente correlacionados aos escores do MEEM, porém, a associação mais forte foi atingida pelo S-TOFHLA (r=0.702, p<0.01) e o S-TOFHLA foi o melhor preditor para os escores do MMSE (R^2^=0.494, p<0.001).

**Conclusões:**

Uma associação mais forte foi alcançada entre os escores no S-TOFHLA e MEEM do que os anos de educação alcançados, este achado pode justificar outros estudos com a incorporação dos anos de escolarização junto aos escores no S-TOFHLA para avaliar o desempenho cognitivo.

Estimates indicate that illiteracy was present in 13.6% of Brazilian population in 2000, falling to 11.8% in 2002, or approximately 14.6 million individuals according to data from the *Instituto Brasileiro de Geografia e Estatística* (IBGE).^1^ In terms of functional illiteracy (defined by the IBGE as less than four years of schooling), the rates vary in different regions, from 19.6% (Southeastern region) to 40.8% (Northeastern region). However, these data probably underestimate illiteracy and functional illiteracy in the rural area of the Northern region. In a previous study, a rate of 97.5% functional illiteracy was identified in a sample of 163 subjects aged 50 years or older, comprising 67.5% illiterates and 30.1% with one to four years of schooling.^2^ According to the IBGE, functional illiteracy affects around 20% of the Brazilian population.

Although mean years of study has risen in recent years,^3^ the mean years of schooling in Brazil in 2000 was 5.7 years, with most functional illiterates being elders with a lower socioeconomic background.^1^

In a cross-sectional study performed in 204 Brazilian cities and evaluating educational status in the elderly, 27% of participants self-reported as being illiterate, 18% had not received any formal education, whereas 22% considered writing and reading a hard task.^4^

Individuals with low educational levels are at higher risk of developing cognitive impairment and dementia.^5^

The Mini-Mental State Examination (MMSE)^6^ has been associated with educational levels by many authors^7-9^ including in Brazil, where numerous studies have verified the influence of schooling on MMSE performance.^10-13^ The MMSE is commonly used for cognitive screening in different settings, as a measure of outcome in clinical trials with preventive and therapeutic drugs, and for therapeutic follow up in clinical practice. The MMSE is recommended as a screening test by the Brazilian Academy of Neurology.^14^

Use of years of schooling to classify a subject’s performance on the MMSE can produce an erroneous result due to the heterogeneity in quality and number of hours spent at school among public and paid learning institutions and to the highly diverse geographic and cultural environments in Brazil. Brucki and Nitrini observed a significant difference in MMSE scores between two different samples (from São Paulo and that of a rural sample from the Amazonian region).^15^ Moreover, number of years of schooling completed is inaccurate because some people continue their education informally. It is not uncommon to encounter individuals with one or two years of formal education, but who are able to comprehend, make inferences or discuss complex subject matter. Conversely, there are also individuals with one or two years or more of formal education that are only able to sign their own name.

Some studies have suggested the use of a reading measure or a literacy test to interpret MMSE scores.^16,17^

Health literacy is defined by the capacity of a person to understand medical information and medical instructions. Literacy level depends on formal educational level attained and on the extent to which people ultimately use reading and writing skills in their daily lives (training). The Test of Functional Health Literacy in Adults (TOFHLA) is an instrument that measures the ability to read and understand medical instructions and health care information.^18^ It has shown good internal consistency, reliability and validity. When individuals with functional illiteracy, or those who cannot read the basic tasks required to function in society, or individuals who have low skills in reading, use the health system, they have significant difficulties with carrying out routine reading tasks such as reading prescriptions of vials of medication, consultation cards, instructions for personal care and health education magazines.^19^ It is important to be able to identify people with limited ability to read, so they can be given special instructions on medications and chronic diseases.

S-TOFHLA is relatively simple and could be useful for determining functional literacy level as well as health literacy level. In a previous study, we sought to verify the degree of correlation between the S-TOFHLA and educational level in Brazil. Carthery-Goulart et al.^20^ verified that the S-TOFHLA was a good measure of literacy in the Brazilian population without cognitive problems, and adaptations made were well accepted. Oliveira et al.^21^ raised the hypothesis that health literacy could be a good measure of literacy among mild cognitive impairment and mild Alzheimer’s disease patients and consequently constitute a reliable measure, together with educational level, for evaluating results on neuropsychological tests. The authors observed that the S-TOFHLA was good for mild cognitive impairment patients, but the test was influenced by greater cognitive impairment, such as in cases of demented patients.

It is a challenge to establish a standard of cognitive normality in a population which is so culturally, educationally, and socioeconomically heterogeneous. The aim of this study was to devise an auxiliary measure to help define different educational level performances.

## Methods

This study was conducted from August 2006 to July 2007, by the team of the Behavioural and Cognitive Neurology Group of the São Paulo University.

An abbreviated version of the Test of Functional Health Literacy in Adults - S-TOFHLA was employed,^19^ which was previously translated and adapted to the Portuguese language. Two independent persons with experience in cognitive evaluation, carried out the translation resulting in a consensual translation, which was back-translated to identify and correct any ambiguous phrases. A detailed explanation of these procedures is described elsewhere.^20^

The study was approved by the Research Ethics Committees of the Hospital das Clínicas of the São Paulo University School of Medicine. All subjects had given written consent for their participation in the study.

### Participants

Subjects were recruited from within the hospital setting (partners of patients, caregivers) and volunteers living in the community that agreed to participate in the study. The subjects were eligible if 18 years of age or older, with at least one year of formal schooling or self-reported informal acquisition of reading skills (subjects with no schooling but able to read were considered as having one year of schooling), and minimum visual acuity of 20/40. Exclusion criteria were any neurological disease or psychiatric disturbances, drug addiction or medical use of any drug with central nervous system action. Uncorrected hearing impairment was an exclusion criteria, as was, the presence of uncontrolled chronic diseases (such as diabetes mellitus, systemic arterial hypertension, etc.). All informants were requested to fill out the Functional Activities Questionnaire^22^ about functionality of any survey participant, and those scoring zero or one point were considered able to continue in the survey.

Participants were submitted to the MMSE.^6,11^ Subsequently, the S-TOFHLA evaluation was performed. The reading comprehension test is comprised by two health-related situations with a total of 36 blank spaces to be filled by one of four possible words from a list. The total score of this part is 72 points. The numeric items consist of cards containing information about medicine intake, date of appointments and results of a laboratory test, and carry a total score of 28 points. The sum of the two parts allow subjects to be classified into three possible literacy conditions: inadequate, borderline, and adequate.

Descriptive analysis was used to describe the sample in terms of age, educational level, scores on the S-TOFHLA and the MMSE. Pearson’s correlation coefficients were used to verify the relationship among years of schooling, S-TOFHLA scores, and MMSE scores. Linear regression was performed with years of schooling, and S-TOFHLA scores as independent variables, and MMSE scores as an dependent variable. Statistical significance was considered at a p-value of 0.05. Analyses were performed using the SPSS 18.0 software package.

## Results

The S-TOFHLA was administered to 325 individuals (207 women/118 men). Demographic characteristics, S-TOFHLA scores and MMSE scores are shown in Table 1.

**Table 1:** Demographics, S-TOFHLA and MMSE scores.

	Minimum value	Maximum value	Median value	Mean	SD
Age (years)	19	81	45	47.13	16.78
Schooling (years)	1	17	11	9.82	5.03
S-TOFHLA scores	0	100	87	76.05	26.38
MMSE scores	19	30	28	27.42	2.39

Strong, positive and correlations between schooling and MMSE scores were found, R=0.536 (p<0.01). However, the most significant positive correlations were between schooling and S-TOFHLA scores, R= 0.736 (p<0.01) and between MMSE scores and S-TOFHLA scores, R=0.702 (p<0.01), proving similar for both genders (Table 2).

**Table 2:** Correlations between MMSE, TOFHLA, and schooling.

	TOFHLA (R)	Schooling (R)
MMSE (total sample)	0.702*	0.536*
MMSE (female)	0.751*	0.589*
MMSE (male)	0.735*	0.497*
Schooling (total sample)	0.736*	-
Schooling (female)	0.736*	-
Schooling (male)	0.708*	-

* p<0.01; R: correlation coefficient (Pearson's correlation).

Multiple linear regression considering education and S-TOFHLA scores as independent variables revealed that all three were predictors of MMSE scores. However taking both education and S-TOFHLA, only the second variable was important in predicting MMSE performance (Table 2). Schooling was responsible for 29.8% of variation in MMSE scores, while the S-TOFHLA was a predictor for 49.3% of variation in the scores, with a minimal difference when taking these two factors together (49.4% of variation).

## Discussion

In this study of 325 community-dwelling subjects, the S-TOFHLA appeared to be a good measure for quantifying health literacy in Brazilian subjects. Our study has demonstrated the usefulness of the S-TOFHLA in predicting performance on the MMSE, performing even better than the schooling variable. In our previous study, age was found not to be an important influence on TOFHLA scores when education was held constant.^20^ The correlation between the total MMSE scores and scores on the S-TOFHLA was much stronger than the relationship between MMSE scores and the number of years of schooling, as previously observed in a sample living in four communities in the USA.^23^

Our sample comprised healthy subjects from different educational backgrounds, representative of our Brazilian population (except for illiterates), whose cognitive evaluation may be diverse according to quality of previous individual schooling and to cultural background.

Based on these findings we can conclude that schooling is responsible for 29.8% of variance in MMSE scores, while the S-TOFHLA score is responsible for 49% of variance in MMSE scores.

Schooling was strongly related to TOFHLA scores, but cannot completely explain these scores. This finding is very interesting because it suggests other factors are influencing the final test score besides formal education. Cultural backgrounds, previous job demands, reading and writing habits, and a genetic factor are all probable factors responsible for the remaining variance.

Other authors have studied the association of S-TOFHLA and cognitive performance. Federman et al. found inadequate health literacy level in 24.3% of 414 participants, and individuals with 1.5 standard deviations or more MMSE scores below age-based norms for the USA population had a six-fold greater adjusted odds of inadequate health literacy. These authors attempted to associate bad performance on cognitive tests and inadequate literacy, although they recognized that the relationship between cognition and health literacy is likely to be bidirectional.^24^

In a systematic review in health literacy, weighted prevalence of low level was 26% and of marginal level was 20% where factors associated with bad performance were level of education and age.^25^ In another study, limited health literacy was predicted by poor self-rated reading ability, more frequently needed help reading written health materials, lower educational level, male gender, and race. It is important to check individuals’ understanding of explanatory material and health information.^26^

Some limitations of our study include that the S-TOFHLA could not be considered a perfect instrument to evaluate functional literacy, although the test does present some items of daily living, such as interpretation of sentences, calculations, and inferences, giving an ecological aspect of evaluation. Our sample was chosen based on convenience; whereby the majority of the participants were caregivers or companions to patients, and thus more prone to cope with hospital information.

Further studies are necessary to determine the influence of health literacy on other cognitive scores, and to establish its efficacy in helping to achieve more reliable cutoff scores with literacy level and schooling, particularly in low educational and literacy levels. Stability of scores on the S-TOFHLA must be determined throughout the aging process, and assessed to confirm whether it is a good measure for predicting performance in cognitively impaired subjects such as mild cognitive impairment and dementia patients.

## Figures and Tables

**Table 3:** Linear regression with MMSE scores as dependent variable.

	R^2^	B	p-value
Education	0.298	0.546	<0.001
S-TOFHLA	0.493	0.702	<0.001
S-TOFHLA and education	0.494	0.6570.061	<0.0010.301

## References

[1] Instituto Brasileiro de Geografia e Estatística (IBGE).

[2] Brucki SMD, Nitrini R (2009). Subjective memory impairment in a rural population with low
education in the Amazon rainforest: an exploratory study. Int Psychogeriatr.

[3] Moreira DA (2003). Analfabetismo funcional: o mal nosso de cada dia.

[4] Neri AL (2007). Idosos no Brasil: vivências, desafios e expectativas na terceira
idade.

[5] Nitrini R, Bottino CMC, Albala C (2009). Prevalence of dementia in Latin America: a collaborative study of
population-based cohorts. Int Psychogeriatr.

[6] Folstein MF, Folstein SE, McHugh PR (1975). Mini-Mental State: a practical method for grading the cognitive
state of patients for the clinician. J Psych Res.

[7] Magaziner J, Bassett SS, Hebel JR (1987). Predicting performance on the Mini-Mental State Examination: use
of age- and education-specific equations. J Am Geriatr Soc.

[8] Bird HR, Canino G, Rubio-Stipec M (1989). Use of Mini-Mental State Examination in a probability sample of
Hispanic population. J Nerv Ment Dis.

[9] Crum RM, Anthony JC, Bassett SS, Folstein MF (1993). Population-based norms for the Mini-Mental State Examination by
age and educational level. JAMA.

[10] Bertolucci PHF, Brucki SMD, Campacci S, Julião Y (1994). O Mini-Exame do Estado Mental em uma população
geral: impacto da escolaridade. Arq Neuropsiquiatr.

[11] Brucki SMD, Nitrini R, Caramelli P, Okamoto IH, Bertolucci PHF (2003). Suggestions for utilization of Mini-Mental State Examination in
Brazil. Arq Neuropsiquiatr.

[12] Laks J, Baptista EM, Contino AL, de Paula EO, Engelhardt E (2007). Mini-Mental State Examination norms in a community dwelling
sample of elderly with low schooling in Brazil. Cad Saude Publica.

[13] Kochhann R, Cerveira MO, Godinho C, Camozzato A, Chaves MLF (2009). Evaluation of Mini-Mental State Examination scores according to
different age and education strata, and sex, in a large Brazilian healthy
sample. Dement Neuropsychol.

[14] Nitrini R, Caramelli P, Bottino CM, Damasceno BP, Brucki SM, Anghinah R (2005). Diagnosis of Alzheimer's disease in Brazil: cognitive and
functional evaluation. Recommendations of the Scientific Department of
Cognitive Neurology and Aging of the Brazilian Academy of
Neurology. Arq Neuropsiquiatr.

[15] Brucki SMD, Nitrini R (2010). Mini-Mental State Examination among lower educational levels and
illiterates: transcultural evaluation. Dement Neuropsychol.

[16] Mayeux EJ, Jr Davis TC, Jackson RH (1995). Literacy and self-reported educational levels in relation to
Mini-Mental State Examination scores. Fam Med.

[17] Baker DW, Gazmarian JA, Sudano J, Patterson M, Parker RM, Williams MV (2002). Health literacy and performance on the Mini-Mental State
Examination. Aging Ment Health.

[18] Williams MV, Parker RM, Baker DW (1995). Inadequate functional health literacy among patients at two
public hospitals. JAMA.

[19] Baker DW, Williams MV, Parker RM, Gazmararian JA, Nurss J (1999). Development of a brief test to measure functional health
literacy. Pat Edu Counsell.

[20] Carthery-Goulart MT, Anghinah R, Areza-Fegyveres R (2009). Performance of a Brazilian population on the test of functional
health literacy in adults. Rev Saude Publica.

[21] Oliveira MO, Porto CS, Brucki SMD (2009). S-TOFHLA in mild Alzheimer's disease and Mild Cognitive
Impairment patients as a measure of functional literacy: preliminary
study. Dement Neuropsychol.

[22] Pfeffer RI, Kurosaki TT, Harrah CH, Chance JM, Filis S (1982). Measurement of functional activities in older adults in the
community. J Gerontol.

[23] Baker DW, Gazmararian JA, Sudano J (2002). Health literacy and performance on the Mini-Mental State
Examination. Aging Ment Health.

[24] Federman AD, Sano M, Wolf MS, Siu AL, Halm EA (2009). Health literacy and cognitive performance in older
adults. J Am Geriatr Soc.

[25] Paasche-Orlow MK, Parker RM, Gazmararian JA, Nielsen-Bohlman LT, Rudd RR (2005). The prevalence of limited health literacy. J Gen Intern Med.

[26] Jeppesen KM, Coyle JD, Miser WF (2009). Screening questions to predict limited health literacy: a
cross-sectional study of patients with diabetes mellitus. Ann Fam Med.

